# Effect of problem-based learning combined with seminar versus traditional teaching method in medical education in China: a systematic evaluation and meta-analysis

**DOI:** 10.3389/fmed.2025.1592199

**Published:** 2025-06-30

**Authors:** Haozhong Wang, Qiang Yuan, Yinling Guo, Xiuli Zheng, Jian Luo, Junhui Qian

**Affiliations:** ^1^College of Basic Medicine, Chengdu University of Traditional Chinese Medicine, Chengdu, Sichuan, China; ^2^Department of Tuina, Hospital of Chengdu University of Traditional Chinese Medicine, Chengdu, Sichuan, China; ^3^Guang’an Hospital of Traditional Chinese Medicine, Guang’an, Sichuan, China

**Keywords:** problem-based learning, seminar, medical education, application effectiveness, meta-analysis

## Abstract

**Objectives:**

This study systematically evaluates the effectiveness of combining problem-based learning with the seminar teaching method and the traditional lecture-based learning model in medical education by meta-analysis.

**Methods:**

A computer-based search of major domestic and international literature databases was conducted, including PubMed, EMBASE, Web of Science Core Collection, Cochrane Library, China National Knowledge Infrastructure(CNKI), Wanfang Database, VIP Chinese Science and Technology Periodicals Database, and China Biology Medicine disk (CBMdisc). The search period spanned from the inception of the databases to 30 August 2024. Quantitative synthesis was performed using the RevMan V.5.4 software, following the Cochrane Reviewer’s Handbook guidelines and the Preferred Reporting Items for Systematic Reviews and Meta-analyses statement.

**Results:**

A total of 13 articles involving 857 medical students were included. The meta-analysis results revealed statistically significant differences between the experimental and control groups in the following areas: theoretical knowledge scores (MD = 4.99, 95% CI: 4.29–5.69, *p* < 0.00001); clinical skill scores (MD = 4.98,95% CI: 4.21–5.75, *p* < 0.00001); case analysis ability (SMD = 3.07, 95% CI: 2.66–3.47, *p* < 0.00001); Learning interest (SMD = 2.46, 95% CI: 1.89–3.03, *p* < 0.00001); Active learning (SMD = 3.26, 95% CI: 2.66–3.85, *p* < 0.00001); teamwork abilities (SMD = 1.66, 95% CI: 1.27–2.05, *p* < 0.00001); students’ research and academic ability (MD = 26.85, 95% CI: 24.79–28.91, *p* < 0.00001). The experimental group demonstrated superior outcomes in all areas compared to the control group.

**Conclusion:**

This meta-analysis showed that the integration of problem-based learning and seminar teaching methods is an effective method for improving theoretical knowledge scores, clinical skill scores, case analysis ability, learning interest, active learning, teamwork abilities and research and academic ability.

## 1 Introduction

In recent years, the field of medical education has undergone significant changes, particularly in teaching methods. The traditional teacher-centered learning and lecture-based learning (LBL) has gradually been replaced by new models focused on students, aiming to enhance their self-directed learning abilities and clinical thinking skills. Among these new teaching methods, problem-based learning (PBL) has been widely applied and studied worldwide due to its unique educational philosophy and approach. PBL fully respects students’ primary roles, helping them learn actively and stimulating their creative thinking (1). Research indicates that PBL can effectively improve students’ exam scores, interest in learning, self-directed learning abilities, and teamwork skills (2). A systematic review and network meta-analysis were conducted to evaluate and rank various teaching strategies in medical education, including simulation-based learning (SBL), flipped classrooms (FC), problem-based learning (PBL), team-based learning (TBL), case-based learning (CBL), and bridge-in, objective, pre-assessment, participatory learning, post-assessment, and summary(BOPPPS). Among these six teaching strategies, CBL and PBL showed greater effectiveness in improving the learning quality and efficiency for medical students (3, 4). Combining PBL with seminars not only improves teaching quality and students’ exam scores but also enhances the overall quality of students’ learning. Despite the many advantages of PBL and its variants in medical education, their application still faces challenges, such as the need for more teaching resources, including time, manpower, and materials (5). PBL demands more from teachers, requiring them to invest additional time in curriculum design and preparation. Teachers need a higher level of professional ability and innovative awareness in designing teaching content and selecting cases. Good organization and management, alongside an effective evaluation mechanism, are needed to ensure the quality of teaching and learning outcomes (6, 7). And PBL, when combined with the seminar technique, is primarily applied to senior students in clinical settings. These students are expected to have the essential basic knowledge needed to understand the topic. The long-term effects and impacts of PBL and its combination with the seminar teaching method require further research and verification. Recently, many reports have emerged on the application of PBL combined with seminars in Chinese medical education, but they all involve small sample sizes, and there currently are no reports on a meta-analysis of its application effects. In light of this, the current study aims to comprehensively evaluate the effects of PBL combined with the seminar teaching method and traditional teaching methods in medical education through systematic evaluation and meta-analysis. By sorting and analyzing the existing literature, we hope to provide a scientific basis for selecting and optimizing teaching models in medical education, thereby promoting the overall improvement of medical education quality and providing a reference for future research.

## 2 Materials and methods

This study protocol has not been previously published. This systematic review and meta-analysis was conducted in accordance with the guidelines outlined in the Cochrane Handbook for Systematic Reviews of Interventions (8) and the Preferred Reporting Items for Systematic Review and Meta-Analysis Protocols (PRISMA-P) (9).

### 2.1 Data sources and search strategy

Two review authors (YQ and WHZ) independently searched eight electronic databases, including PubMed, EMBASE, Web of Science Core Collection, Cochrane Library, China National Knowledge Infrastructure (CNKI), Wanfang Database, VIP Chinese Science and Technology Periodicals Database, and China Biology Medicine disk (CBM disk), from the inception of the databases to 30 August 2024, without language restrictions, to identify relevant studies. We used the following combined text and MeSH terms: “Problem-Based Learning” and “Seminar Teaching” and “Education, Medical.” The complete search used for PubMed was: [“Problem-Based Learning” (Mesh)] OR [Learning, Problem-Based (Title/Abstract)] OR [Problem Based Learning (Title/Abstract)]) OR [Curriculum, Problem-Based (Title/Abstract)] OR [Curriculum, Problem Based (Title/Abstract)] OR [Problem-Based Curriculum (Title/Abstract)] OR [Problem-Based Curricula (Title/Abstract)] OR [Curricula, Problem-Based (Title/Abstract)] OR [Problem Based Curricula (Title/Abstract)] OR [Experiential Learning (Title/Abstract)] OR [Learning, Experiential (Title/Abstract)] OR [Active Learning (Title/Abstract)] OR [Learning, Active (Title/Abstract)] AND (Seminar Teaching) AND [(“Education, Medical) (Mesh)] OR [Medical Education (Title/Abstract)]. Corresponding modifications were made to meet the requirements of the other databases, and all potentially eligible studies were considered for review. Additionally, a manual search was performed using the reference lists of key articles published in English, with any discrepancies resolved by consulting a third reviewer (QJH).

### 2.2 Eligibility criteria

The retrieved research was considered eligible when it fulfilled the predefined inclusion criteria as follows: (1) Studies with medical students as subjects, applying the PBL + seminar teaching method to medical courses; (2) Included studies are randomized controlled trials (RCTs); (3) Intervention measures such as the control group uses the traditional LBL teaching method, while the experimental group uses the PBL + seminar teaching method; and (4) Objective outcome measures such as theoretical knowledge scores or Clinical skills scores to evaluate learning effectiveness. Studies were excluded if one of the following conditions was met: (1) The literature lacks usable data, (2) Duplicate publications, (3) Non-medical majors, or (4) Non-compliance with RCT criteria.

### 2.3 Study selection and data extraction

Two researchers (WHZ and YQ) independently conducted literature screening and information extraction, and cross-checked each other’s work. In cases of disagreement, a third researcher (LJ) decided whether to include the study. The content included in the review consists of the first author, publication date, sample size, discipline and course, student characteristics, course content, baseline data comparison, and observed outcome variables.

### 2.4 Risk of bias assessment

Two evaluators (QJH and LJ) independently used the RevMan tool for randomized trials to categorize the risk of bias as high, unclear, or low across the following seven domains: random sequence generation (selection bias), allocation concealment (selection bias), blinding of participants and personnel (performance bias), blinding of outcome assessment (detection bias), incomplete outcome data (attrition bias), selective reporting (reporting bias), and other biases. A third evaluator (WHZ) was consulted to resolve inconsistencies.

### 2.5 Statistical analysis

The included studies were analyzed using the RevMan 5.4 software for meta-analysis. Continuous data were expressed using the weighted mean difference and 95% confidence interval (CI) as effect size indicators. The X^2^ test was employed to analyze the heterogeneity of the results among studies. If the heterogeneity among the study results was not statistically significant (I^2^ < 50%, *p* > 0.10), a fixed-effects model was used to create a forest plot. If heterogeneity was statistically significant (I^2^ > 50%, *p* < 0.10), a random-effects model was used to create a forest plot. Significant heterogeneity was addressed using subgroup analysis, sensitivity analysis, or descriptive analysis only. The level of significance for meta-analysis was set at α = 0.05. When more than 10 studies were included, a funnel plot was used to assess publication bias. Sensitivity analysis was conducted by sequentially omitting individual studies to evaluate the robustness of the combined effects.

## 3 Results

### 3.1 Literature search results

**FIGURE 1:**
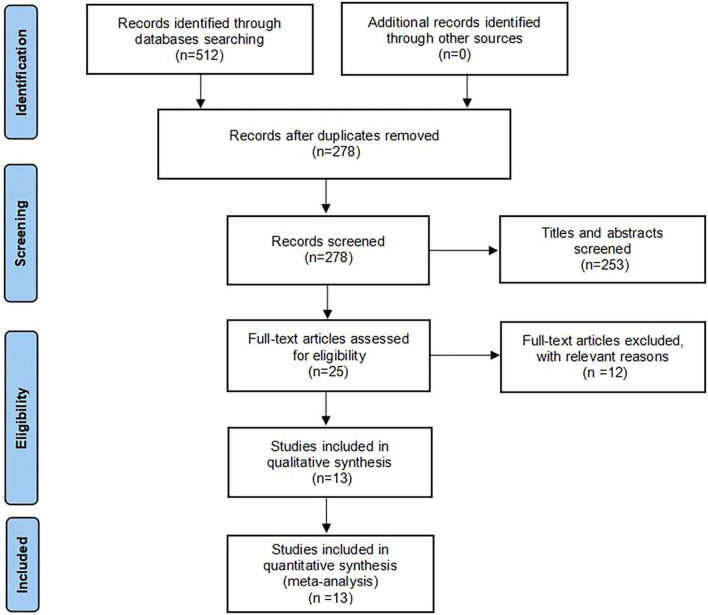
The Preferred Reports for Systematic Reviews and Meta-Analyses (PRISMA) flow diagram for the literature search and exclusion criteria.

Initially, 512 studies were identified by using a predefined search strategy. After excluding duplicates, 278 studies were included in the analysis. Upon initial review, 253 articles unrelated to medical research education, or non-empirical in nature, were excluded. Subsequently, 25 remaining articles were screened in full text, and ultimately 13 eligible studies [Ji et al. (10), Ye et al. (11), Li and Peng (12), Zhou et al. (13), Wang et al. (14), Xia et al. (15), Lei et al. (16), Wu et al. (17), Wu et al. (18), Gao and Hu (19), Li et al. (20), Sun et al. (21), Zhang et al. (22)] involving a total of 857 participants were included in this meta-analysis. A PRISMA flow diagram of the literature search and exclusion criteria is shown in Figure 1.

### 3.2 Data characteristics

This meta-analysis included 13 RCTs involving 857 participants (435 in the PBL combined with seminar education group and 422 in the traditional LBL control group). All the studies were conducted in China and published between 2017 and 2024, all in Chinese. PBL combined with the seminar teaching method was clearly defined in all studies. The basic characteristics of the included studies are presented in Table 1.

**TABLE 1:** Detailed baseline characteristics of all included studies.

References	Publication time	No. of PBL combined with seminar	No. of LBL	Students	Course name	Baseline data comparison	Outcome measures
Ji et al. (10)	2017	15	15	Undergraduate	Oral and maxillofacial surgery	Unmentioned	①②
Ye et al. (11)	2018	50	52	Undergraduate	Hematology	*P* > 0.05	①②
Li and Peng (12)	2020	25	25	Undergraduate	Ultrasonic medicine	*P* > 0.05	①
Zhou et al. (13)	2020	38	36	Undergraduate	Social medicine	*P* > 0.05	①
Wang et al. (14)	2021	16	16	Postgraduate	Nephrology	*P* > 0.05	①②③
Xia et al. (15)	2021	38	38	Resident physician	Urology	*P* > 0.05	①②
Lei et al. (16)	2022	48	45	Associate degree student	Tradition Chinese medicine	*P* > 0.05	①②
Wu et al. (17)	2019	66	56	Resident physician	Critical care medicine	*P* > 0.05	①②③
Wu et al. (18)	2022	24	24	Postgraduate	Reproductive medicine	*P* > 0.05	①④⑤⑤⑦
Gao and Hu (19)	2023	30	30	Resident physician	TCM rehabilitation	*P* > 0.05	①②③⑤⑦⑦
Li et al. (20)	2023	30	30	Postgraduate	Pulmonology	*P* > 0.05	①⑦
Sun et al. (21)	2023	36	35	Postgraduate	Clinical medicine	*P* > 0.05	①②
Zhang et al. (22)	2023	19	20	Undergraduate	Gastroenterology	*P* > 0.05	①②④

No., number; PBL, problem-based learning; LBL, lecture-based learning;①, theoretical knowledge;②, clinical skills;③, case analysis;④, learning interest;⑤, active learning;⑦, teamwork abilities;⑦, research and academic ability.

### 3.3 Bias risk and evidence quality

**FIGURE 2:**
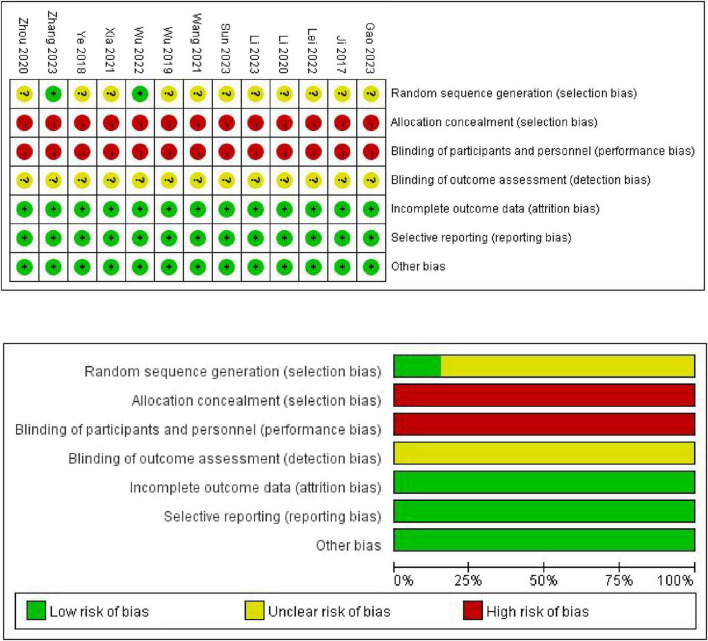
Risk of bias summary for each included study.

The Cochrane bias risk tool for assessing the risk of randomized trials revealed that all 13 studies mentioned randomization, but only two [Wu et al. (18), Zhang et al. (22)] described specific methods of random allocation. As all studies were educational, allocation concealment and blinding of participants and personnel were not applicable. All studies had complete data; thus, the attrition bias was assessed as low risk. All studies had a low risk of incomplete outcome data, selective reporting, and other biases. A summary of the risk of bias for each study is presented in Figure 2.

## 4 Meta-analysis results

### 4.1 Theoretical knowledge scores

**FIGURE 3:**
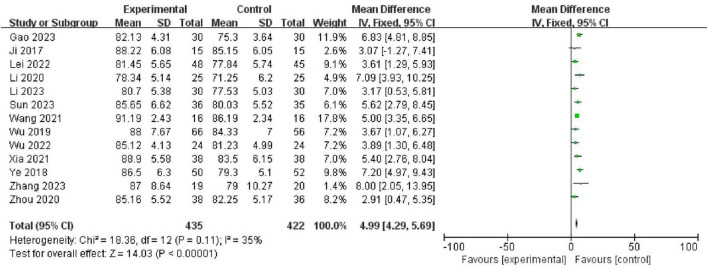
Forest plot for effectiveness of problem-based learning (PBL) + seminar versus lecture-based learning (LBL) in theoretical knowledge scores.

A total of 13 studies [Gao and Hu (19), Ji et al. (10), Lei et al. (16), Li and Peng (12), Li et al. (20), Sun et al. (21), Wang et al. (14), Wu et al. (17), Wu et al. (18), Xia et al. (15), Ye et al. (11), Zhang et al. (22), Zhou et al. (13)] compared theoretical knowledge scores involving 857 participants (435 in the PBL + seminar group, and 422 in the LBL group). Using the fixed-effects model, the meta-analysis results showed that the theoretical knowledge scores of students in the PBL + seminar group were significantly higher than those in the traditional group (LBL), (MD = 4.99, 95% CI: 4.29–5.69, *p* < 0.00001). A forest plot is shown in Figure 3.

### 4.2 Clinical skill scores

**FIGURE 4:**
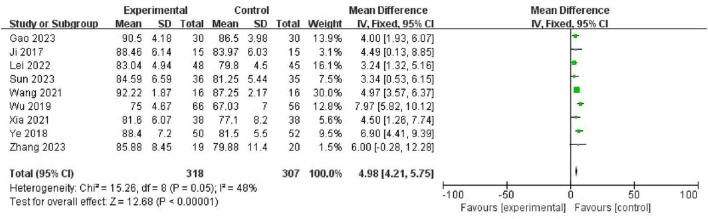
Forest plot for effectiveness of problem-based learning (PBL) + seminar versus lecture-based learning (LBL) in clinical skill scores.

Six studies [Gao and Hu (19), Ji et al. (10), Lei et al. (16), Sun et al. (21), Wang et al. (14), Wu et al. (17), Xia et al. (15), Ye et al. (11), and Zhang et al. (22)] reported clinical skill scores involving 625 participants (318 in the PBL + seminar group and 307 in the LBL group). Using the fixed-effects model, the meta-analysis results illustrated that clinical skill scores of students in the PBL + seminar group were significantly higher than those in the traditional group (LBL), (MD = 4.98, 95% CI: 4.21–5.75, *p* < 0.00001). A forest plot is shown in Figure 4.

### 4.3 Comprehensive abilities

**FIGURE 5:**
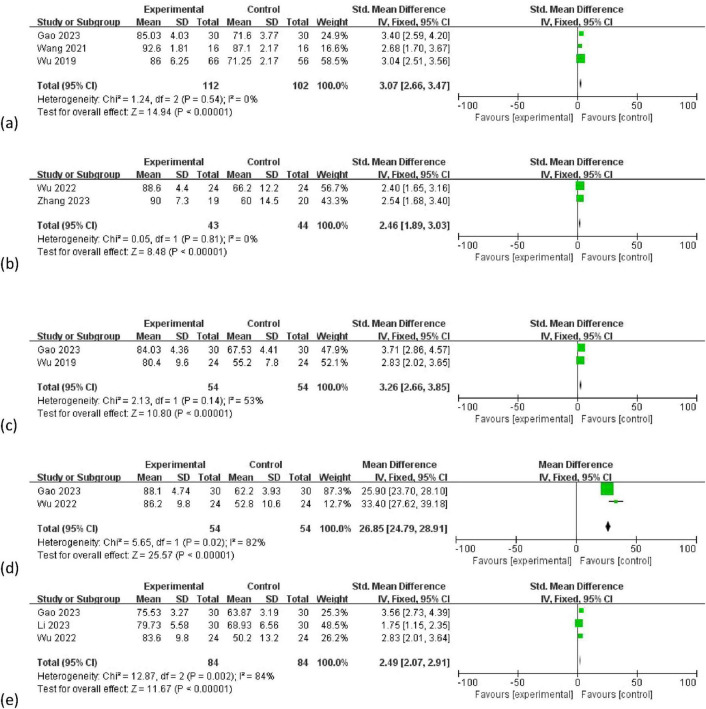
Forest plot for effectiveness of problem-based learning (PBL) + seminar versus lecture-based learning (LBL) in **(a)** case analysis ability; **(b)** learning interest **(c)** active learning ability **(d)** teamwork ability **(e)** research and academic ability.

Three studies [Gao and Hu (19), Wang et al. (14), Wu et al. (17)] assessed 214 students (112 in the PBL + seminar group, 102 in the LBL group) and found that the PBL + seminar method significantly improved case analysis abilities (SMD = 3.07, 95% CI: 2.66–3.47, *p* < 0.00001) (Figure 5a). Two studies [Wu et al. (18), Zhang et al. (22)] involving 87 students (43 in the PBL + seminar group and 44 in the LBL group) showed it increased learning enthusiasm (SMD = 2.46, 95% CI: 1.89–3.03, *p* < 0.00001) (Figure 5b). Three studies [Gao and Hu (19), Wu et al. (17)] with 108 students (54 in each group) indicated it stimulated self-directed learning (SMD = 3.26, 95% CI: 2.66–3.65, *p* < 0.00001) (Figure 5c). Three studies [Gao and Hu (19), Wu et al. (17)] involving 179 students (90 in the PBL + seminar group and 89 in the LBL group) found it enhanced teamwork abilities (MD = 26.85, 95% CI: 24.79–28.91, *p* < 0.00001) (Figure 5d). Three studies [Gao and Hu (19), Li et al. (20), Wu et al. (18)] with 168 students (84 in each group) showed it improved research and academic abilities (SMD = 2.49, 95% CI: 2.07–2.91, *p* < 0.00001) (Figure 5e).

Subgroup analyses further identified that variations in course implementation methods contributed to heterogeneity. Specifically Gao and Hu (19), Wu et al. (18) focused on medical information retrieval, where PBL-seminar sessions emphasized database searching and critical appraisal of literature. This likely enhanced students’ research skills (I^2^ = 54% for this subgroup).

### 4.4 Assessment of publication bias

**FIGURE 6:**
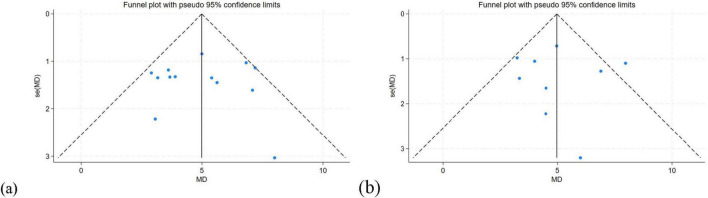
(a) Funnel plot of knowledge scores. **(b)** Funnel plot of clinical skill scores.

A funnel plot was used to assess the publication bias. The results showed that the theoretical knowledge and Clinical skill scores were symmetrical, indicating no publication bias (Figures 6a, b). Egger’s test of knowledge scores showed *P* = 0.922 (Table 2a), and Egger’s test of clinical skill scores showed *P* = 0.903. Both *P*-values exceed 0.05 (Table 2b), indicating no evidence of publication bias.

**TABLE 2a:** Egger’s test of knowledge scores.

Std_eff	Coefficient	Std. err.	t	*P* > |t|	(95% conf. interval)	
Slope	5.159415	1.735837	2.97	0.013	1.338863	8.979967
Bias	−0.135803	1.35349	−0.10	0.922	−3.114814	2.843208

**TABLE 2b:** Egger’s test of clinical skill scores.

Std_eff	Coefficient	Std. err.	t	*P* > |t|	(95% conf. interval)	
Slope	4.787557	1.601816	2.99	0.020	0.9998636	8.575251
Bias	0.17178	1.360622	0.13	0.903	−3.04558	3.38914

## 5 Discussion

Currently, various teaching methods focused on PBL are widely applied in the medical field. PBL is a commonly used teaching method in medical education, and is a student-centered education approach based on real-world scenarios. PBL guides students in exploring and solving problems independently through the presentation of specific problems, thereby promoting the development of their critical thinking and self-directed learning abilities (7). However, teachers did not participate in student discussions. Although the questions raised by students can trigger some thinking among their peers, owing to their limited knowledge reserve, students’ answers may be subject to varying degrees of questioning, which affects the construction of thinking patterns for problems and can easily lead to misunderstandings or deviations from the topic.

Seminar, characterized mainly by discussion-based teaching, focus on interactions between teachers and students. Under the guidance of teachers, students discuss and learn about specific topics, which is a method of gradually cultivating students’ ability to engage in scientific research independently, actively discover learning problems, and formulate solutions. The seminar teaching method can be summarized as “pre-class preparation and research, in-class reporting and discussion, and post-class thesis formation” for these three processes (23). Although all the research collected in the database focuses on China, it does not mean that the conclusions of the report are geographically limited. We believe they are also applicable to other regions. The combination of PBL and seminar can enhance students’ self-directed learning abilities, independent thinking skills, communication skills, analytical and problem-solving abilities, and teamwork capabilities (24). Through group discussions and case analyses, students’ interest in the learning content increases, and learning becomes more active and in-depth (25). The combination of PBL with seminar helps students combine theoretical knowledge with clinical practice, fostering clinical thinking and problem-solving skills (21). The seminar segment encourages interactions between teachers and students, allowing teachers to better understand students’ learning conditions and provide timely feedback (26). The process of group discussion and collaborative problem solving helps cultivate students’ team spirit and collaborative skills (18). Students are generally more satisfied with PBL combined with the seminar teaching method, and they prefer this interactive approach; through case discussions, students can better understand and apply knowledge, enhancing their practical clinical application skills. Although PBL-seminars are beneficial, they also face practical challenges like needing more teaching resources or teacher training. Here are some suggestions to overcome these obstacles: First, increase teaching resources by raising funds to buy equipment and teaching materials. Second, strengthen teacher training through regular sessions, expert guidance, and case sharing. Third, cooperate with other schools to share resources and training opportunities, reducing costs and improving efficiency.

## 6 Limitations

Although this study mentioned RCTs, only some of the studies described the method of random grouping, and none mentioned whether blinding was used. This may be because it is difficult to implement double-blind experiments in teaching research. Significant heterogeneity was found in the combined analysis of multiple outcome indicators, which may have resulted from differences in factors such as educational background, academic level, academic major, teaching materials, course content, types of students, and students’ subjective feelings about teaching satisfaction. In addition, there was considerable publication bias in the questionnaire scoring outcome indicators of the included studies, which may be related to the low quality of the studies. These limitations could affect the generalizability of the conclusions of this meta-analysis. however, they did not affect the authenticity of the results. Future studies should employ rigorous randomization techniques like simple or stratified random sampling and detail the randomization process.

## 7 Conclusion

In conclusion, the present study suggests that PBL combined with seminar is more effective than LBL for medical education. The combination of PBL and seminar teaching method not only improves scores in theoretical knowledge exams, clinical skill exams, but also enhances students’ case analysis ability, interest in learning, their proactive learning abilities, teamwork skills, and research and academic capabilities. This approach has been positively recognized by the students. However, due to limited data and the low quality of the methodologies of the included studies, more well-designed, high-quality RCTs with larger sample sizes are warranted to confirm whether PBL combined with the seminar approach is superior to traditional teaching methods in China.

## Data Availability

The original contributions presented in this study are included in this article/supplementary material, further inquiries can be directed to the corresponding authors.
